# Beyond Accuracy: Reaction Time and Task Format Effects in Early Mathematical Processing

**DOI:** 10.3390/bs16071086

**Published:** 2026-07-01

**Authors:** Yuan-Horng Lin, I-Ping Wan

**Affiliations:** 1 Department of Mathematics Education, National Taichung University of Education, Taichung 403060, Taiwan; lyh@mail.ntcu.edu.tw; 2 Graduate Institute of Linguistic, National Chengchi University, Taipei 116011, Taiwan; 3 Research Center for Mind, Brain and Learning, National Chengchi University, Taipei 116011, Taiwan

**Keywords:** early mathematics learning, reaction time, accuracy, gender-related variation, mathematical cognition, contextualized mathematical problems, mixed-effects modeling

## Abstract

This exploratory study explores associations among task format, gender-related variation, response accuracy and reaction time (RT) in early mathematics learning. Since traditional assessments often emphasize correctness, the study combines accuracy and RT measures to capture children’s temporal processing patterns during problem solving. Thirty-seven first-grade students in Taiwan (21 girls, 16 boys; M_age = 7.2 years, SD = 0.43) completed two computerized tasks implemented using PsychoPy/Pavlovia involving formula-based symbolic arithmetic and competency-based problems with contextualized text and visual representations. Mixed-effects models were used to analyze RT and accuracy at participant and item levels. Accuracy was higher in the formula-based condition than in the competency-based condition. The Experiment × Gender interaction was not significant, and the final accuracy model showed a significant effect of experiment but no evidence of a significant gender effect. Boys showed numerically higher accuracy than girls in the formula-based condition, but this difference was reduced in the competency-based condition. For correct trials, RTs were longer in the competency-based condition; neither gender nor the Experiment × Gender interaction was significant. In the all-trial RT model, significant Accuracy × Experiment and Accuracy × Gender interactions indicated that RT differences between correct and incorrect trials varied by condition and gender. The fixed task order requires cautious interpretation of these findings.

## 1. Introduction

In recent years, research on early mathematical cognition has increasingly emphasized the role of representational format in shaping how young learners access and process numerical information, particularly within technology-mediated learning environments. In digital and computer-based instructional contexts, representational format has become a design variable that can be systematically manipulated through interfaces, multimedia elements and presentation structures. For instance, Ho et al. (2022) and Chiu et al. (2025) have emphasized how mathematics teaching and learning are increasingly shaped by technology-mediated environments, ranging from virtual classroom implementations to the design of interactive learning tasks. These studies demonstrate the need to examine how learners engage with different task designs and representations in digital contexts in addition to assessing learning outcomes.

A substantial body of research on early arithmetic and word-problem solving has shown that children’s performance is strongly influenced by whether mathematical content is presented in abstract symbolic form or embedded in linguistic and visual contexts (e.g., Bjork & Bowyer-Crane, 2013; Bruner, 1974; Cummins et al., 1988; Duval, 2006; Jitendra et al., 2015; Mayer, 2009a; Nathan et al., 1992; Paivio, 1991; Swanson et al., 2008; Thevenot et al., 2007). These representational differences are particularly consequential in technology-enhanced learning settings, where visual layout, text density and multimodal integration directly shape learners’ cognitive engagement.

From a cognitive perspective, differences between symbolic and contextualized representations reflect fundamental constraints on working memory, symbolic mapping and the integration of language with numerical information in the developing mind (Sweller, 1988; Duval, 2006; Cowan, 2014). Symbolic mathematics requires children to translate learned numerical symbols (such as Arabic numerals and operators) into quantity meanings while coordinating rules and intermediate steps through executive control (Baddeley, 2000; Geary, 2011). For young learners, the mapping between symbolic forms and underlying meanings remains unstable and effortful, as numerical symbols have not yet become automatized and, therefore, require deliberate linkage to quantity representations (Schneider & Shiffrin, 1977; Kersey et al., 2018). In technology-mediated tasks, these demands can be further shaped by interface complexity and representational density. Consequently, formula-based problems may impose substantial cognitive demands, particularly for first-grade students whose executive functions and symbolic fluency are still developing (Cowan, 2014; Kail, 1991; Mayer & Moreno, 2003; Sweller, 1988). By contrast, although text/image competency-based problems require additional reading and semantic integration, reaction time does not necessarily increase uniformly, as semantic and perceptual cues in these formats can support meaning construction and allow some children to rely on context-driven or heuristic strategies (Cummins et al., 1988; Nathan et al., 1992; Thevenot et al., 2007). Dual Coding theory further explains why text/image-based tasks may support early mathematical processing. Information presented through coordinated verbal and visual channels can facilitate integration and retrieval by engaging multiple forms of representation (Mayer, 2009b; Paivio, 1991). This account is consistent with developmental research showing that young children often benefit from contextualized word problems, as semantic narratives and visual cues can help them construct relational models before translating them into formal operations (Carpenter et al., 1999; Verschaffel et al., 2000). Such support may be especially important in early elementary grades when symbolic competence is still developing and constrained by working memory and attention (Cowan, 2014; Geary, 2011).

Early mathematical learning has traditionally been evaluated primarily through accuracy, with less attention to the temporal characteristics of children’s responses. In technology-enhanced learning environments, reaction time provides an additional behavioral indicator of processing efficiency and task demands. Longer response times may reflect less automatized processing or greater cognitive effort even when accuracy remains relatively intact (Kail, 1991; Schneider & Shiffrin, 1977). Although meta-analytic findings have reported broad similarity in overall mathematics performance during childhood, particularly at the elementary school level (Hyde et al., 1990), studies of reaction time and strategy use suggest that more subtle variation may appear in processing dynamics and speed–accuracy patterns across task conditions (Halpern et al., 2007). Such variation may be especially observable in early grades, when language skills, executive control and symbolic fluency are still developing. Therefore, RT may help reveal how children respond to the differing demands of formula-based and text/image competency-based tasks.

Large-scale international assessments such as PISA (OECD, 2018) and TIMSS (IEA, 2020) have consistently shown that students’ mathematical performance depends on the representational, linguistic and contextual formats in which problems are presented. Whereas TIMSS primarily assesses students’ mastery of curriculum-based and symbolically expressed mathematical procedures, PISA places greater emphasis on learners’ ability to apply mathematical reasoning and problem solving in real-world, language- and context-rich situations. These assessment contrasts highlight how representational and linguistic design choices in digital tasks can differentially affect learners’ processing dynamics. Therefore, the present study focuses on first-grade students in a Taiwan Mandarin-speaking context, where instructional materials are presented in Traditional Chinese characters. It examines how young learners solve computer-based mathematics problems in two task formats: formula-based procedural items and text/image competency-based tasks. Using trial-level accuracy and reaction-time data, the study analyzes how task format and gender-related variation are associated with children’s processing patterns in a touch-based digital learning environment.

Drawing on Cognitive Load theory (Sweller, 1988) and Dual Coding theory (Mayer, 2009b; Paivio, 1991), the present study examines how mathematical task format is associated with early learners’ accuracy and reaction time during computer-based problem solving. Although international research has investigated task characteristics in mathematical problem solving, relatively few studies have examined how reaction time and accuracy jointly vary across formula-based and text/image competency-based tasks, particularly among early elementary learners. In Taiwan, empirical studies examining first-grade learners’ processing patterns in digital mathematics environments remain limited, with existing research relying primarily on paper-based assessments or self-report measures. Using trial-level data and mixed-effects modeling, this study investigates task-format effects and gender-related patterns at both participant and item levels. The study addressed the following research questions.

(1) To what extent do Grade 1 learners show different accuracy and reaction-time patterns across formula-based and text/image competency-based mathematical tasks?
(2) How are trial-level accuracy and reaction time associated across different mathematical task formats?
(3) To what extent do response-time and accuracy patterns vary across gender groups under different mathematical task conditions?

To address these research questions, the present study conducts two computerized cognitive tasks implemented in PsychoPy/Pavlovia to examine how first-grade learners process formula-based arithmetic tasks and text/image competency-based tasks. This study contributes to existing RT and accuracy research by examining trial-level processing patterns in first-grade learners during computer-based mathematical problem solving. Correctness and reaction time are used as complementary indicators, and gender is included as a learner characteristic to examine task-specific and context-dependent variation across formula-based procedural tasks and text/image competency-based tasks in a Taiwan Mandarin-speaking instructional context.

## 2. Materials and Methods

Although the sample size was relatively limited, the within-subject design and trial-level analyses allowed the study to examine variation in processing patterns across different task formats in early mathematical learning. Thirty-nine first-grade students from two mixed-gender public elementary school classrooms in Taichung, Taiwan, were initially recruited to participate in the study. One student was absent on the day of testing, and another completed only Experiment 1. As a result, data from 37 students (21 girls, 16 boys; M_age = 7.2 years, SD = 0.43) were included in the final analyses, yielding a total of 1110 recorded responses across all tasks. All participants were native Taiwan-Mandarin speakers. Participation was voluntary, and written parental consent was obtained in accordance with ethical guidelines approved by the affiliated institution. Participants received a small stationery gift set and a gift voucher equivalent to NT$400 as compensation for participation, regardless of task performance or completion status.

Participants were tested in small groups during regular school hours in a classroom setting. All tasks were administered using the PsychoPy/Pavlovia platform 2025.1.0. (Peirce et al., 2022) on identical tablet devices (iPadOS 18.4) through the Safari browser, ensuring consistency in hardware and software across participants. Stimuli were presented in a standardized web-based format, and responses were made by selecting one of four multiple-choice options through a touch-screen interface. The system automatically recorded response accuracy and response time in milliseconds at the trial level. Children were instructed to complete the tasks comfortably without explicit emphasis on speed or accuracy and were informed that the activity was not an examination. Prior to data collection, the experimental procedure and platform functionality were tested with adult participants to ensure technical stability. In addition, the task materials were reviewed by experienced teachers to ensure age appropriateness for first-grade learners.

The experiment employed two mathematics problem formats to contrast symbolic and context-based processing in early arithmetic. The order of the two experimental conditions was fixed, with all participants completing Exp 1 prior to Exp 2. However, item presentation within each condition was fully randomized, such that the presentation order differed across participants. The fixed order of the two conditions was adopted for practical and pedagogical reasons. The participants were first-grade students who had received only about nine months of formal mathematics instruction. In the elementary school curriculum, formula-based arithmetic is typically introduced before more reading-intensive and context-based mathematical problems. Presenting the competency-based condition first was therefore considered less appropriate for these young learners and might have disrupted the familiar instructional progression used in their classroom context. In addition, administering different task orders within the same classroom setting would have increased procedural complexity for teachers and could have raised concerns among parents regarding differences in testing conditions. For these reasons, a fixed order was used to maintain feasibility, classroom consistency and ecological validity in the school-based assessment context.

**Figure 1:**
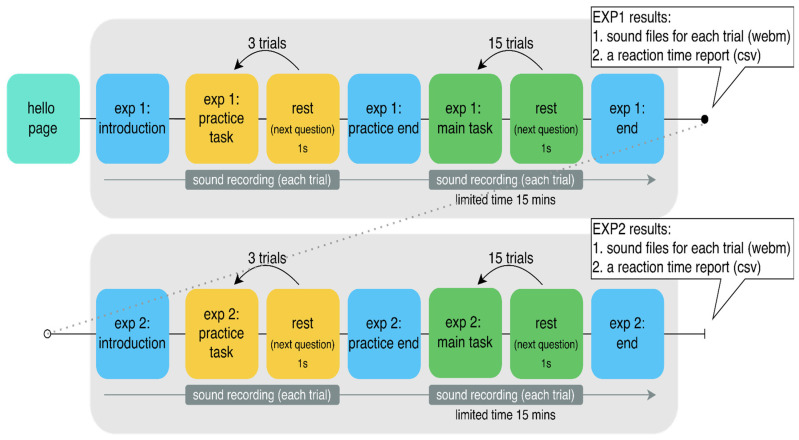
Workflow of the experimental procedure.

In the formula-based condition (Exp 1), students solved conventional arithmetic expressions such as 3 + 5 = ?, which required processing abstract numerical symbols and procedural computation. In the text/image competency-based condition (Exp 2), students solved real-world word problems presented in traditional Chinese characters with accompanying Taiwan phonetic symbols (Zhuyin Fuhao; e.g., 繁體中文, ㄈㄢˊ ㄊㄧˇ ㄓㄨㄥ ㄨㄣˊ, “traditional Chinese”) and visual illustrations (e.g., a problem equivalent to “There are fifteen eggs. If eight eggs are used to make a cake, how many eggs are left?”), which required the integration of linguistic information and pictorial cues prior to calculation. In addition, the mathematics items used in this study were aligned with the Grade 1 curriculum and focused on foundational number sense and basic addition and subtraction in Taiwan. These included counting, comparing quantities, understanding ones and tens and solving simple arithmetic problems. Each experimental condition included 18 items, consisting of 3 practice trials and 15 target items. Practice trials were presented at the beginning of each condition to familiarize participants with the task format and interface and were excluded from all analyses. The remaining target items were presented in randomized order and included in the main analyses. The full set of experimental materials for both experiments is provided in Appendix A. The web-based online platform PsychoPy/Pavlovia has been used to provide technical support for the precise measurement of reaction times and the logging of task-related behavioral patterns. The experimental workflow is illustrated in Figure 1.

The order of experimental conditions was fixed, with Experiment 1 preceding Experiment 2, while item presentation within each condition was randomized. Classroom teachers confirmed that children with reading difficulties did not participate in the study. In addition, all task materials were reviewed by experienced teachers before data collection to ensure that the wording, instructions and visual presentation were age-appropriate for first-grade students. Recording durations were constrained (approximately 15 min per task) to minimize fatigue and maintain consistency across participants. Practice trials were excluded from all analyses. Reaction time data from both correct and incorrect trials were processed using the same preprocessing pipeline. Trials with missing responses or non-positive RT values were removed prior to analysis, and RT data were log-transformed to reduce skewness. RTs beyond ±2.5 SDs from each participant’s mean were also excluded. The resulting statistical patterns were consistent with the original analyses.

All analyses were conducted in R (version 4.6.0) using lme4 (version 2.0.1) for mixed-effects modeling (Bates et al., 2015), lmerTest for Satterthwaite-adjusted *p*-values (Kuznetsova et al., 2017), emmeans for post hoc comparisons, performance for model diagnostics and ggplot2 for visualization. Mixed-effects models included random intercepts for participants and items to account for variability across individuals and stimuli. Random slopes were not included because more complex models resulted in convergence or singularity issues given the relatively limited sample size. Accuracy data were analyzed using generalized linear mixed-effects models (GLMMs) with a binomial logit link function, whereas reaction time data were analyzed using linear mixed-effects models (LMMs) on log-transformed RTs. Fixed effects included experiment, gender and their interaction where appropriate, with random intercepts for participants and stimuli. Full model formulas and statistical specifications are available in the OSF repository listed in the Data Availability Statement. The following models were fitted:

Accuracy model (GLMM, binomial logit):

accuracy ~ experiment + gender + (1 | participant_id) + (1 | stimulus_name)

Reaction time model (LMM, log-transformed RT):

log_rt ~ experiment × gender + (1 | participant_id) + (1 | stimulus_name)

Accuracy–RT model (GLMM):

accuracy ~ log_rt + experiment × gender + (1 | participant_id) + (1 | stimulus_name)

RT–accuracy model (LMM):

log_rt ~ accuracy × experiment × gender + (1 | participant_id) + (1 | stimulus_name)

## 3. Results

**Figure 2:**
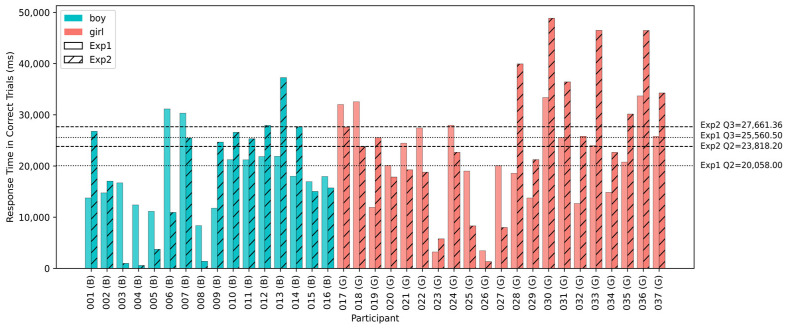
Correctness × RT in Formula-Based versus Competency-Based tasks.

In this study, descriptive analyses and distributional plots were used to visualize performance patterns and error distributions. The results first present descriptive statistics, followed by mixed-effects model results for accuracy and reaction time, analyses of the relationship between accuracy and reaction time, and finally a summary relative to the research questions. Figure 2 illustrates participant-level correctness rates across the two experimental conditions by gender with distributional summaries.

Figure 2 shows response times for correct trials by participant, gender and experimental conditions (Exp 1: formula-based; Exp 2: text/image competency-based). Each bar represents an individual participant’s proportion of correct responses, with green bars indicating boys and red bars indicating girls. Solid bars correspond to Exp 1, whereas hatched bars correspond to Exp 2. Dashed horizontal lines indicate group mean accuracy. At the group level, both boys and girls showed longer response times in the text/image competency-based condition than in the formula-based condition. At the gender level, boys in Exp 1 showed a mean reaction time of 19.2 s for correct responses, whereas in Exp 2 their mean reaction time increased to 24.8 s. Girls also exhibited slower responding in Exp 2. In Exp 1, girls’ mean reaction time for correct responses was 23.4 s, whereas in Exp 2 it increased substantially to 30.9 s. Although the descriptive increase appeared larger for girls, individual-level patterns varied across participants. Following the accuracy analyses, Table 1 provides an overview of individual response patterns across the two experimental conditions.

**Figure 3:**
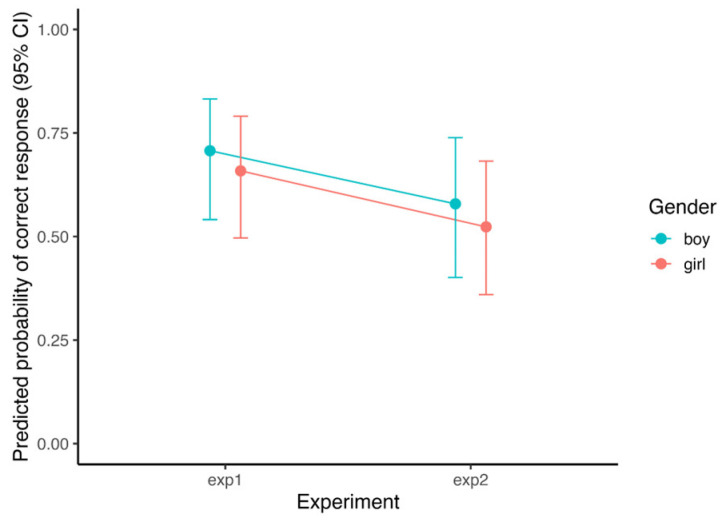
Accuracy × Experiments × Gender.

Note that RTs beyond ±2.5 SDs from each participant’s mean were excluded before the descriptive statistics were calculated. As shown in the table, the cleaned data reveal gender-related variation in performance between the two experimental conditions. A higher proportion of correct responses for boys is observed in the formula-based condition (Exp 1), whereas correctness distributions are more comparable between gender groups in the text/image competency-based condition (Exp 2). At the item level, boys answered 150 out of 223 items correctly in Exp 1, corresponding to an accuracy rate of 67.3%. In Exp 2, boys answered 108 out of 194 items correctly, yielding an accuracy rate of 55.7%. For girls, 178 out of 291 items were answered correctly in Exp 1, corresponding to 61.2% accuracy. In Exp 2, girls answered 147 out of 278 items correctly, corresponding to 52.9% accuracy. Although boys show a higher proportion of correct responses than girls in the formula-based condition, this gender-related pattern narrows in the competency-based condition, where both groups exhibit lower overall accuracy and more comparable correctness distributions. A GLMM with a binomial link function was used to analyze response accuracy. Experimental condition and gender were entered as fixed effects, and random intercepts were included for participants and items. The intercept indicates the baseline log-odds of a correct response in Exp 1, as shown in Figure 3.

**Figure 4:**
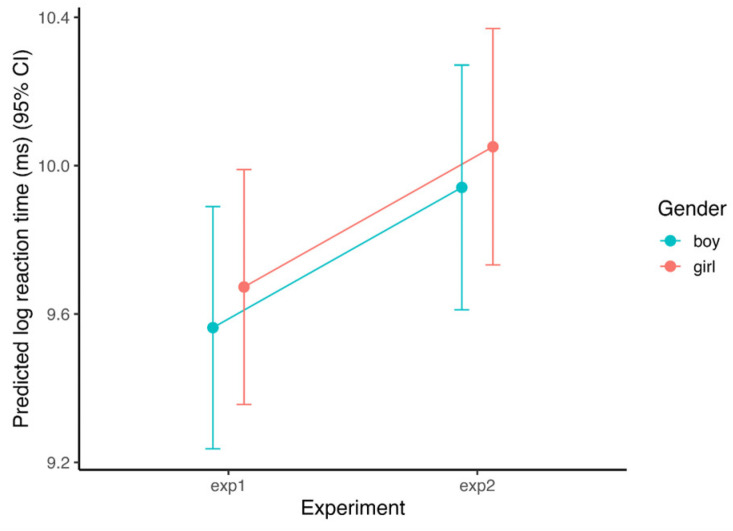
RT × Experiment × Gender.

As shown in Figure 3, the predicted probability of a correct response was higher in Exp 1 than in Exp 2 for both gender groups. Accuracy was analyzed using a GLMM with a binomial distribution, with experiment and gender as fixed effects and random intercepts for participants and stimuli. An initial model including the Experiment × Gender interaction showed no significant interaction; therefore, the additive model was retained for reporting. The final model showed a significant effect of experiment. Accuracy in Exp 2 was lower than in Exp 1 (β = −0.563, SE = 0.151, z = −3.72, *p* < 0.001), corresponding to reduced odds of a correct response in Exp 2 (OR = 0.57, 95% CI [0.42, 0.77]). Pairwise comparison confirmed that accuracy was significantly higher in Exp 1 than in Exp 2 (OR = 1.76, 95% CI [1.31, 2.36], z = 3.724, *p* < 0.001). Girls showed numerically lower predicted accuracy than boys in Exp 1 (Girl: M = 0.66, 95% CI [0.50, 0.79]; Boy: M = 0.71, 95% CI [0.54, 0.83]), but the additive model showed no significant main effect of gender once experimental condition and random effects were taken into account (β = −0.225, SE = 0.344, z = −0.65, *p* = 0.514). The LMM analysis of log-transformed reaction times further showed substantial variability across participants and items, as shown in Figure 4.

**Figure 5:**
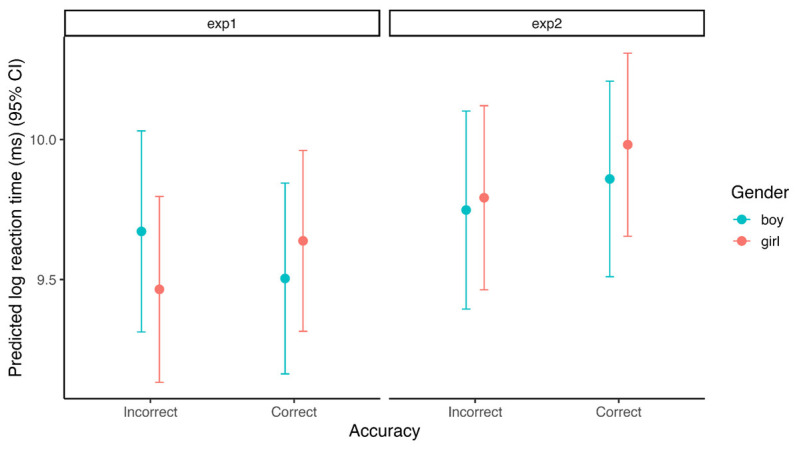
RT × Accuracy × Experiment × Gender.

Log-transformed RTs for correct trials were longer in Exp 2 than in Exp 1. A likelihood ratio test indicated that the Experiment × Gender interaction was not significant, χ^2^(1) = 0.011, *p* = 0.917, and therefore, the interaction term was removed and the additive model was retained. The additive model showed a significant effect of experiment (β = 0.378, SE = 0.052, t = 7.29, *p* < 0.001), with pairwise comparisons confirming longer log RTs in Exp 2 than in Exp 1 (estimate = 0.378, 95% CI [0.276, 0.480], t = 7.275, *p* < 0.001). Model-estimated marginal means showed higher predicted log RTs in Exp 2 than in Exp 1 for both boys (Exp 1: M = 9.56, 95% CI [9.24, 9.89]; Exp 2: M = 9.94, 95% CI [9.61, 10.27]) and girls (Exp 1: M = 9.67, 95% CI [9.36, 9.99]; Exp 2: M = 10.05, 95% CI [9.73, 10.37]). The main effect of gender was not significant (β = 0.110, SE = 0.115, t = 0.96, *p* = 0.349). Figure 5 displays the EMMs with 95% confidence intervals, accounting for participant- and item-level variability.

The all-trial RT model examined whether the association between trial accuracy and log-transformed RT varied by experiment and gender. The model included Accuracy (Incorrect vs. Correct), Experiment (Exp 1 vs. Exp 2), Gender (boy vs. girl), and their interactions as fixed effects with random intercepts for participants and items. The main effect of Accuracy was not statistically significant (β = −0.168, t = −1.65, *p* = 0.100), and the main effects of Experiment and Gender were also not statistically significant (Experiment: t = 0.71, *p* = 0.480; Gender: t = −1.22, *p* = 0.228). However, these relationships were not uniform across groups. Among two-way interactions, Accuracy × Experiment was significant (β = 0.279, SE = 0.138, t = 2.02, *p* = 0.044), as was Accuracy × Gender (β = 0.341, SE = 0.131, t = 2.61, *p* = 0.009). The Experiment × Gender interaction was not significant (β = 0.251, SE = 0.138, t = 1.82, *p* = 0.069). Figure 5 illustrates these model-estimated patterns using predicted log RTs with 95% confidence intervals across accuracy levels, experiments and gender groups. The model-estimated pattern indicated that correct trials had longer predicted RTs than incorrect trials in Exp 2, and that girls showed longer predicted RTs for correct than incorrect trials.

The three-way interaction among Accuracy, Experiment and Gender was not statistically significant (β = −0.263, SE = 0.179, t = −1.47, *p* = 0.142), indicating that the RT–accuracy association did not differ reliably by both experiment and gender. Follow-up comparisons for the significant Accuracy × Experiment interaction showed that, in Exp 2, log RTs were significantly longer for correct than incorrect trials (estimate = 0.150, 95% CI [0.014, 0.287], t = 2.161, *p* = 0.031), whereas this difference was not significant in Exp 1 (estimate = 0.003, 95% CI [−0.137, 0.131], t = 0.039, *p* = 0.969). Follow-up comparisons for the significant Accuracy × Gender interaction further showed that girls had significantly longer log RTs for correct than incorrect trials (estimate = 0.181, 95% CI [0.056, 0.307], t = 2.833, *p* = 0.005), whereas this difference was not significant for boys (estimate = −0.029, 95% CI [−0.179, 0.122], t = 0.372, *p* = 0.710). These findings suggest that RT–accuracy associations varied by experimental condition and gender group without supporting a stable gender-specific effect.

The findings address the research questions at three levels. First, Grade 1 learners showed lower accuracy in Exp 2 than in Exp 1, whereas the accuracy model showed no evidence of a significant gender effect or Experiment × Gender interaction. Gender-related patterns in accuracy should therefore be interpreted as descriptive trends since the model did not show a significant gender effect or Experiment × Gender interaction. Second, correct-trial RTs were longer in Exp 2 than in Exp 1, with no significant gender effect or Experiment × Gender interaction. Third, the all-trial RT model showed that RT–accuracy associations varied by experimental condition and gender group. Although the Accuracy × Experiment × Gender interaction was not significant, the significant Accuracy × Experiment interaction showed that correct trials were associated with longer RTs than incorrect trials in Exp 2 but not in Exp 1. The significant Accuracy × Gender interaction further indicated a gender-specific modulation of the RT–accuracy association, in which girls showed longer RTs for correct than incorrect trials, whereas this contrast was not significant among boys. These results are consistent with condition- and gender-related modulation of RT–accuracy associations, but do not support a stable gender-specific effect. Given the fixed task order, however, the condition-related findings should be interpreted cautiously and should not be attributed to task format alone.

## 4. Discussion

The present study examined how accuracy and reaction time jointly characterize early mathematical task performance in first-grade learners under two experimental conditions. The results indicate that differences between the formula-based and text/image competency-based conditions were not limited to accuracy outcomes but also extended to temporal response patterns. In particular, longer reaction times were generally observed in the text/image competency-based condition. Given the fixed task order adopted for school-based and pedagogical reasons, however, these differences should be interpreted as condition-related patterns and would not be attributed to task format alone. Reaction time is not interpreted here as a direct proxy for cognitive load or strategy use. Instead, it is treated as an index of temporal processing that reflects how learners engaged with different task characteristics under the present testing conditions. The observed relationships between response time and accuracy varied across conditions and do not correspond to a classic speed–accuracy trade-off. These findings suggest that accuracy and response time provide complementary information about early mathematical task performance.

In the present study, girls showed a stronger observed association between accuracy and longer response times in the text/image-based condition, whereas boys showed less differentiation in response time as a function of accuracy. The observed gender-related patterns appear to be closely tied to task format and should be interpreted cautiously within the present exploratory framework. The findings suggest that response-time measures may help reveal condition-specific processing patterns that are not apparent from accuracy outcomes alone. Such patterns may emerge during early mathematics learning, particularly when children move from formula-based symbolic problems to context-rich text/image competency-based tasks.

The present study further highlights the need to interpret gender-related patterns in early mathematics learning with caution. Some prior studies with older students or adults have reported advantages for girls on text- or context-based problems and faster responding for boys on symbolic tasks. The present findings suggest that such expectations may not apply straightforwardly to Grade 1 learners. For young children, context-rich mathematical problems may involve multiple task characteristics, including linguistic complexity, semantic integration, visual information, working memory and inhibitory control. These characteristics may contribute to longer response times and more differentiated RT–accuracy associations under some testing conditions. In the present study, the accuracy model did not show a significant gender effect or Experiment × Gender interaction, and the correct-trial RT model likewise showed no significant gender effect or Experiment × Gender interaction. Gender-related patterns were more evident in the all-trial RT analysis, where the Accuracy × Gender interaction indicated that girls showed longer RTs for correct than incorrect trials, a pattern that was not observed reliably among boys. These findings suggest that response-time measures may reveal condition- and gender-related variation in RT–accuracy associations beyond what is captured by accuracy outcomes. This interpretation remains constrained by the fixed task sequence, which prevents a clear separation of task-format effects from order-related influences.

In addition, prior research has also suggested that gender-related variation in mathematics is more likely to emerge in relation to task characteristics and problem-solving approaches, whereas broad performance differences are less consistently found. Meta-analytic evidence indicates that overall gender-related differences in mathematics achievement are small or negligible, and observed variability is often attributable to task characteristics (Lindberg et al., 2010). Other studies have emphasized gender-related variation in problem-solving strategies and solution patterns even when accuracy differences are less consistently observed (e.g., Zhu, 2007; Gallagher et al., 2000). This literature provides a useful context for the present exploratory findings, in which gender-related patterns were not supported in the accuracy model but emerged in the RT–accuracy analysis. The results, therefore, suggest that reaction time may provide complementary information about task-specific performance patterns beyond overall accuracy comparisons.

In terms of processing dynamics, the present results highlight the value of reaction time as an additional process-level measure for describing how young learners engage with different task formats. Accuracy and reaction time did not map onto each other in a uniform way, as similar levels of performance were associated with different temporal profiles. These patterns suggest that temporal response profiles varied with task condition and trial accuracy. In the all-trial RT analysis, the significant Accuracy × Experiment and Accuracy × Gender interactions indicated that the timing difference between correct and incorrect trials varied by experimental condition and gender group. Correct trials were associated with longer RTs in Exp 2 but not in Exp 1, and girls showed longer RTs for correct than incorrect trials, a pattern that was not observed reliably among boys. These findings suggest that young learners’ temporal response patterns may vary with task characteristics, including linguistic, contextual, symbolic and visual features. At the same time, the observed patterns should be understood as condition-specific variation in RT–accuracy relationships within the present exploratory design, especially given the fixed task sequence.

The present findings highlight the value of incorporating process-sensitive measures when examining early mathematical task performance. Accuracy captures outcome-level performance, while reaction time provides complementary information about how learners respond to different mathematical task conditions during problem solving. The inclusion of temporal measures, therefore, contributes to a more nuanced understanding of early mathematics learning, particularly in context-rich tasks where linguistic, symbolic and visual information must be integrated. At the same time, the interpretation of these temporal patterns takes into account the fixed order of task administration. Because the formula-based condition always preceded the text/image competency-based condition, the observed differences between conditions cannot be separated from potential order-related influences, including fatigue, practice or familiarization with the response interface, or changes in motivation. The longer reaction times observed in the text/image competency-based condition may therefore reflect task-related differences, order-related influences, or both. Accordingly, the reaction-time findings should be interpreted as condition-related patterns within the present school-based design.

## 5. Conclusions

The present study examined accuracy and reaction time in first-grade learners under two mathematical task conditions. The results showed that formula-based and text/image competency-based tasks were associated with different performance patterns, with differences observed not only in accuracy but also in reaction time. RT–accuracy associations further varied across task conditions and gender groups, suggesting that temporal response profiles may provide information that is not fully captured by accuracy outcomes. These findings highlight the value of incorporating reaction time as a complementary process-level measure in studies of early mathematical cognition. At the same time, the findings should be interpreted cautiously given the relatively small sample size, the exploratory nature of the study and the fixed order of task administration. The study contributes to ongoing discussions regarding task format, temporal processing, and gender-related variation in early mathematics learning, and suggests that process-sensitive measures may offer useful perspectives for future research on digital learning environments and early mathematical cognition.

Several limitations should be acknowledged. First, the present analyses focused on first-grade learners and relied primarily on reaction time as an index of temporal processing during mathematical problem solving. Although reaction time provides a useful temporal measure of task engagement, it does not directly capture the full range of cognitive, affective or strategic processes involved in children’s responses. In addition, the relatively small sample size limits the stability of interaction estimates, particularly for gender-related patterns. A major limitation of the present study concerns the fixed order of the experimental conditions. All participants completed the formula-based condition before the text/image competency-based condition. Therefore, the task format was fully confounded with task order. The observed differences between Experiments 1 and 2 should not be interpreted as effects of task format only, because they may also reflect order-related factors such as practice, fatigue, familiarization with the tablet-based response interface, motivational changes, or other procedural effects. This limitation is particularly relevant for the interpretation of reaction time, which may be sensitive not only to task characteristics but also to changes in attention, motivation or familiarity over the course of testing. Future studies should counterbalance the order of task conditions or use a randomized design to separate task-format effects from order-related influences. The two task formats also differed along multiple dimensions, including linguistic complexity, contextualization, visual representation and task difficulty, which cannot be fully disentangled within the present experimental design. Furthermore, the study did not include independent measures of cognitive abilities such as working memory, reading proficiency or attentional control; therefore, interpretations regarding underlying cognitive mechanisms remain tentative.

To address some of these limitations, a third experiment has been designed as a preference-based task to complement the present reaction-time analyses. In this task, children participated in structured interviews that were audio- and video-recorded during the experimental procedure. Voice recordings are being analyzed using Praat/Parselmouth to extract acoustic energy-related parameters, and video recordings collected during the PsychoPy/Pavlovia-based procedure are being examined through facial-feature extraction. In ongoing analyses, additional vocal features are also being explored using emo2vec to further characterize affective and prosodic patterns associated with mathematical engagement. This multimodal design may provide complementary process-level evidence in addition to reaction time and allow for a richer characterization of how children engage with different mathematical task contexts. Future research with larger samples, more controlled task manipulations, and multimodal analytical approaches will help clarify how task characteristics, instructional design and affective engagement jointly shape early mathematical processing. Such work may also contribute to the development of adaptive learning environments that better support diverse learning needs during early mathematics education.

## Figures and Tables

**Table 1:** Gender × Correctness.

Exp 1: Gender × Correctness				
Gender/Correctness	TRUE	%	FALSE	%
Boy	150	67.3%	73	32.7%
Girl	178	61.2%	113	38.8%
Exp 2: Gender × Correctness				
Gender/Correctness	TRUE	%	FALSE	%
Boy	108	55.7%	86	44.3%
Girl	147	52.9%	131	47.1%

## Data Availability

The anonymized datasets and analysis scripts used for the GLMM/LMM analyses in this study are publicly available through the Open Science Framework (OSF) repository. Data sharing was approved by the Institutional Review Board, and all shared materials comply with participant confidentiality and data protection requirements. https://osf.io/62yj8/ (accessed on 16 June 2026).

## Appendix A

Note that the English text below reflects the original meaning and was not shown in the experimental test items.

(1) Experiment 1: Formula-based performance task.

**Pretest**
i. 在空格中填入正確的數字，17 − ____ = 9 Fill in the blank with the correct number: 17 − ____ = 9 (1) 6 (2) 7 (3) 8 (4) 9
ii. 下面哪一個選項的答案，和 9 + 5 的答案一樣？ Which of the following options has the same answer as 9 + 5? (1) 5 + 9 (2) 6 + 9 (3) 9 + 6 (4) 8 + 5
iii. 下面的選項中，哪一個答案是 12？ Which of the following options has the answer 12? (1) 8 + 4 (2) 8 + 5 (3) 9 + 4 (4) 9 + 5
**Main test**
1. 15 − 8 = ? (1) 5 (2) 6 (3) 7 (4) 8
2. 下列哪一個算式的答案，與 7 + 6 相同？ Which of the following equations has the same answer as 7 + 6? (1) 8 + 4 (2) 9 + 4 (3) 6 + 8 (4) 10 + 4
3. 請選出從小到大排列的數字： Please choose the numbers arranged from smallest to largest: (1) 42, 58, 37, 69 (2) 25, 18, 39, 47 (3) 10, 90, 50, 30 (4) 16, 23, 47, 57
4. 2、4、6、___、10，缺少的數是？ 2, 4, 6, ___, 10. What number is missing? (1) 5 (2) 7 (3) 8 (4) 9
5. 數字 94 的個位數字是？ What is the ones digit of the number 94? (1) 49 (2) 9 (3) 4 (4) 94
6. 18 − 6 = ? (1) 9 (2) 10 (3) 12 (4) 11
7. 下列哪一個算式的答案正確？ Which of the following equations is correct? (1) 15 − 6 = 11 (2) 7 + 6 = 12 (3) 6 + 9 = 12 (4) 15 − 8 = 7
8. 下面哪個數最大？ Which of the following numbers is the largest? (1) 78 (2) 85 (3) 65 (4) 92
9. 在空格中填入適當的數字，98、___、96、95、___、___ Fill in the blanks with appropriate numbers: 98, ___, 96, 95, ___, ___ (1) 97, 94, 93 (2) 99, 92, 91 (3) 98, 94, 93 (4) 98, 95, 94
10. 數字 76 的十位數字是？ What is the tens digit of the number 76? (1) 6 (2) 7 (3) 76 (4) 67
11. 在空格中填入正確的答案，47 = ____ − 11 Fill in the blank with the correct answer: 47 = ____ − 11 (1) 57 (2) 58 (3) 59 (4) 68
12. 下列哪一個算式的答案，與 15 − 9 相同？ Which of the following equations has the same answer as 15 − 9? (1) 14 − 8 (2) 11 − 6 (3) 10 − 3 (4) 19 − 11
13. 在 53 和 67 之間，哪個數最接近 60？ Between 53 and 67, which number is closest to 60? (1) 56 (2) 62 (3) 66 (4) 59
14. 在空格中填入適當的數字，49、___、43、40、___、34 Fill in the blanks with appropriate numbers: 49, ___, 43, 40, ___, 34 (1) 48, 36 (2) 46, 37 (3) 45, 38 (4) 47, 38
15. 在 89 和 67 之間，哪個數最接近 74？ Between 89 and 67, which number is closest to 74? (1) 81 (2) 68 (3) 88 (4) 66

(2) Experiment 2: Text/image competency-based performance task.

**Pretest**
i. 小明原本有 9 顆糖果，他現在有 17 顆。請問媽媽給了他幾顆糖果？ Xiaoming originally had 9 candies. Now he has 17 candies. How many candies did his mom give him? (1) 6 (2) 7 (3) 8 (4) 9
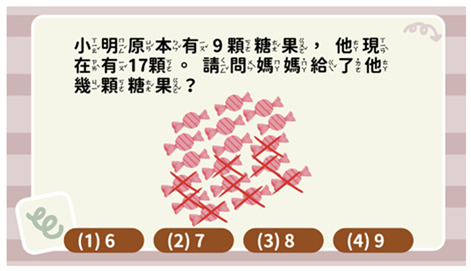
ii. 小明買了 5 顆蘋果，又買了 9 顆，他現在有多少顆蘋果？選出正確的算式！ Xiaoming bought 5 apples and then bought 9 more apples. How many apples does he have now? Choose the correct equation. (1) 5 + 9 (2) 6 + 9 (3) 9 + 6 (4) 8 + 5
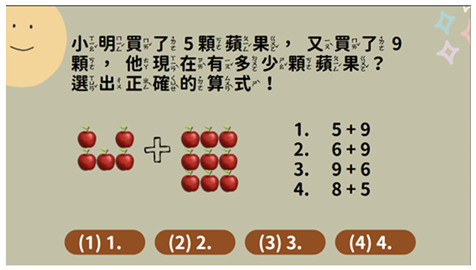
iii. 有 12 片餅乾，其中(___)片是巧克力的，剩下(___)片是奶油的，請選出正確的算式 There are 12 cookies. Among them, (___) are chocolate cookies, and the remaining (___) are butter cookies. Please choose the correct equation. (1) 8 + 4 (2) 8 + 5 (3) 9 + 4 (4) 9 + 5
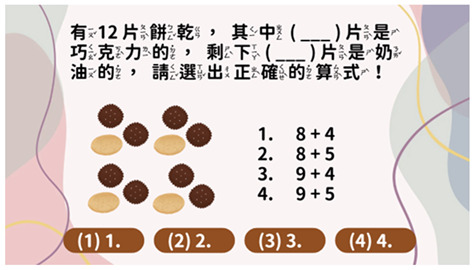
**Main test**
1. 家裡有 15 顆雞蛋，做蛋糕用了 8 顆雞蛋，請問現在還剩下幾顆雞蛋？ There are 15 eggs at home. Eight eggs are used to make a cake. How many eggs are left now? (1) 5 (2) 6 (3) 7 (4) 8
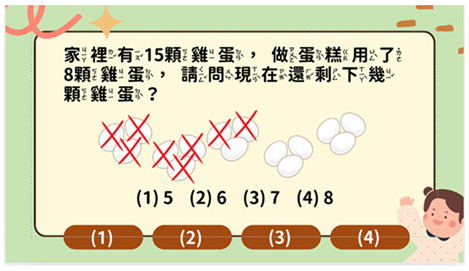
2. 小狗有 7 根骨頭，又找到 6 根，它現在有幾根骨頭？請選出跟答案相同的算式： A puppy has 7 bones and finds 6 more bones. How many bones does it have now? Please choose the equation that has the same answer. (1) 8 + 4 (2) 9 + 4 (3) 6 + 8 (4) 10 + 4
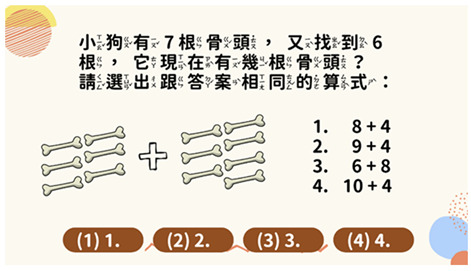
3. 媽媽買了 47 顆草莓，爸爸買了 16 顆，姊姊買了 23 顆，哥哥買了 57 顆。請由少到多，排出大家買的草莓數量： Mom bought 47 strawberries, Dad bought 16, Sister bought 23, and Brother bought 57. Please arrange the numbers of strawberries they bought from the smallest to the largest. (1) 42, 58, 37, 69 (2) 25, 18, 39, 47 (3) 10, 90, 50, 30 (4) 16, 23, 47, 57
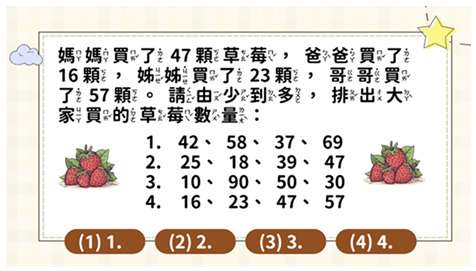
4. 小火車從 10 號站台出發，每經過一站，站台號碼會變小。請填上缺少的站台號碼！2、4、6、___、10 A small train starts from platform number 10. Each time it passes a station, the platform number becomes smaller. Please fill in the missing number: 2, 4, 6, ___, 10. (1) 5 (2) 7 (3) 8 (4) 9
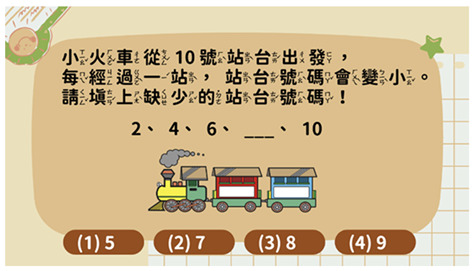
5. 工廠有 94 位工人，請問工人數量的「個位數字」是多少？ There are 94 workers in a factory. What is the ones digit of the number of workers? (1) 49 (2) 9 (3) 4 (4) 94
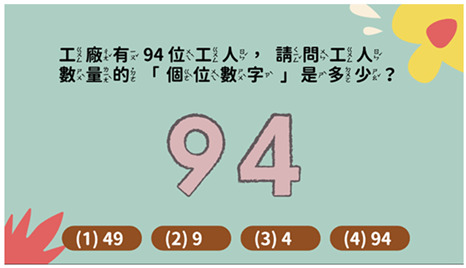
6. 姊姊有 18 塊餅乾，分給妹妹 6 塊，請問現在姊姊還剩下幾塊餅乾？ Sister has 18 cookies and gives 6 cookies to her younger sister. How many cookies does she have left? (1) 9 (2) 10 (3) 12 (4) 11
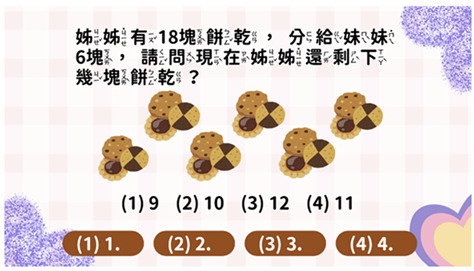
7. 農夫想知道現在有幾隻小鴨在池塘裡？走了幾隻，還剩幾隻？但他算術不好，請幫助農夫選出正確的算式。 A farmer wants to know how many ducklings are in the pond now. Some ducklings walked away. He is not good at math. Please help the farmer choose the correct equation. (1) 15 − 6 = 11 (2) 7 + 6 = 12 (3) 6 + 9 = 12 (4) 15 − 8 = 7
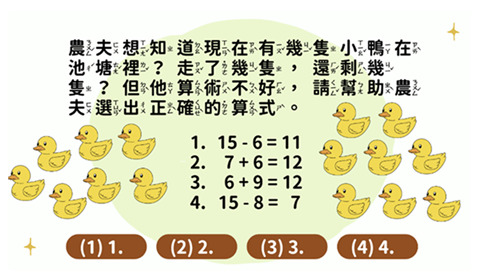
8. 農場裡有四隻小羊，他們分別抓到很多隻小雞。請問他們最多能抓到幾隻小雞？ There are four little lambs on the farm. Each of them catches many chicks. What is the greatest number of chicks they could have caught? (1) 78 (2) 85 (3) 65 (4) 92
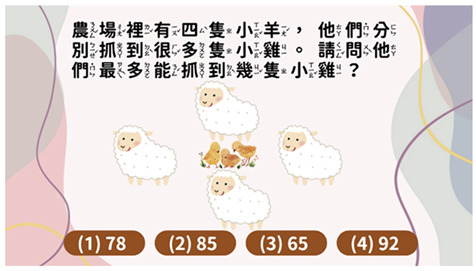
9. 小鳥從 98 號樹飛出去，每飛過一棵樹都會停下來休息。但是，觀察的人發現，紀錄表上有些地方不見了！請你幫忙填上正確的數字：98、___、96、95、___、___ A little bird flies away from tree number 98. Each time it passes a tree, it stops to rest. However, some numbers are missing in the record. Please help fill in the correct numbers: 98, ___, 96, 95, ___, ___. (1) 97, 94, 93 (2) 99, 92, 91 (3) 98, 94, 93 (4) 98, 95, 94
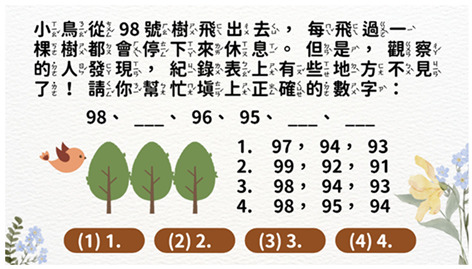
10. 數字 76 的十位數字是？ What is the tens digit of the number 76? (1) 6 (2) 7 (3) 76 (4) 67
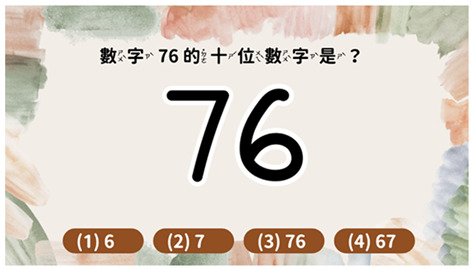
11. 姊姊有很多彈珠，其中 11 顆是紅色的，剩下的 47 顆是藍色的，請問姊姊有幾顆彈珠？ Sister has many marbles. Eleven of them are red, and the remaining 47 are blue. How many marbles does she have in total? (1) 57 (2) 58 (3) 59 (4) 68
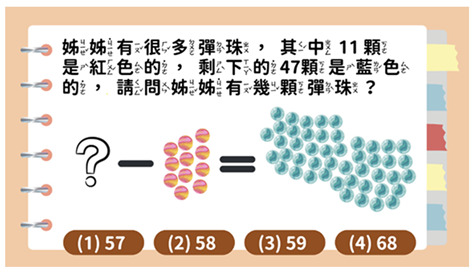
12. 有 15 顆氣球，被風吹走 9 顆。請問下面哪個算式的答案，和剩下的氣球數量一樣？ There are 15 balloons. Nine balloons are blown away by the wind. Which of the following equations has the same answer as the number of balloons left? (1) 14 − 8 (2) 11 − 6 (3) 10 − 3 (4) 19 – 11
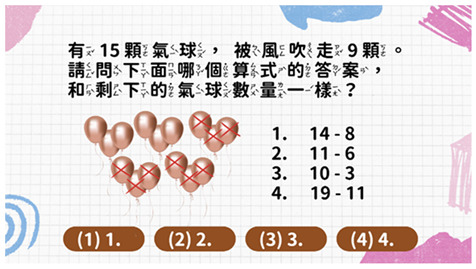
13. 現在紅燈有 53 秒，綠燈有 67 秒，請問紅燈和綠燈的秒數之間，哪個最接近 60 秒？ The red light lasts for 53 s, and the green light lasts for 67 s. Between these two times, which number is closest to 60 s? (1) 56 (2) 62 (3) 66 (4) 59
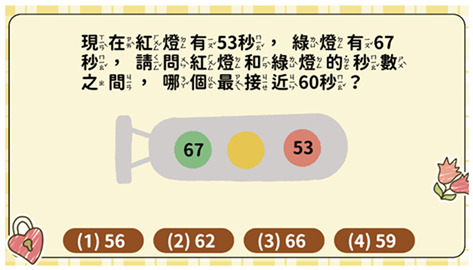
14. 鯨魚從大海裡浮出水面。船長想要帶大家一起看鯨魚，鯨魚出現的位置是：55、52、49…43、40…。請幫助船長先生，找出鯨魚們接下來出現的位置！49、___、43、40、___、34 Whales come up from the ocean. The captain wishes to show the whales to everyone. The whales appeared at the positions: 55, 52, 49… 43, 40…. Please help the captain find the next positions where the whales will appear: 49, ___, 43, 40, ___, 34. (1) 48, 36 (2) 46, 37 (3) 45, 38 (4) 47, 38
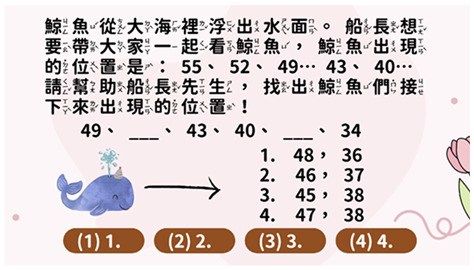
15. 爺爺 89 歲，叔公 67 歲，奶奶 74歲，請問其他家族成員中，誰的年齡跟奶奶最接近？ Grandpa is 89 years old, Uncle is 67 years old, and Grandma is 74 years old. Among the other family members, whose age is closest to Grandma’s? (1) 81 (2) 68 (3) 88 (4) 66
